# Editorial: The foundational components and elements of plant foods for neurological nutrition and well-being

**DOI:** 10.3389/fnut.2026.1904762

**Published:** 2026-07-07

**Authors:** Chuanfeng Tang, Jianmei Li, Yemin Wang

**Affiliations:** 1 State Key Laboratory on Technologies for Chinese Medicine Pharmaceutical Process Control and Intelligent Manufacture, Nanjing University of Chinese Medicine, Nanjing, China; 2 School of Food Science and Pharmaceutical Engineering, Nanjing Normal University, Nanjing, China; 3 Department of Molecular Oncology, British Columbia Cancer Research Centre, Vancouver, BC, Canada; 4 Department of Pathology and Laboratory Medicine, University of British Columbia, Vancouver, BC, Canada

**Keywords:** mechanisms, neurological disorders, neurological health, nutrients, phytochemicals

In the contemporary milieu, conventional “medicinal supplements” are increasingly perceived as an unhealthy approach. Instead, the embrace of “food supplements” by the masses is gaining traction. An accumulating body of evidence underscores the crucial role of specific nutrients and compounds within the diet in maintaining the health and optimal functionality of the nervous system (1, 2). Rich in polyphenols, flavonoids, polysaccharides, dietary fiber, and essential micronutrients, certain foods are recognized for their ability to preserve cardiovascular and cerebrovascular health, combat inflammation and oxidative stress, and prevent neurodegeneration, thereby promoting overall nervous system wellbeing (3–5). Therefore, adopting a healthy dietary regimen substantially reduces the incidence of nervous system disorders, concurrently bolstering nervous system resilience and vitality.

The objective of this Research Topic is to investigate the potential therapeutic benefits of dietary phytoactive compounds, including edible mushrooms, condiments, Chinese herbal medicines, fruits, and nuts, in addressing neurological disorders such as depression, Anxiety, Alzheimer's disease (AD), Parkinson's disease (PD), migraine, epilepsy, vascular cognitive impairment (VCI), and chemotherapy-induced peripheral neuropathy (CIPN). The neuroprotective properties of these phytochemicals have been shown to directly impact the nervous system, thereby inhibiting the onset and progression of neurological conditions. Additionally, they contribute to maintaining nervous system homeostasis and safeguarding neurological health through indirect mechanisms, such as inflammation inhibition and cardiovascular protection. The articles collected in this Research Topic collectively illuminate the multi-target mechanisms by which plant-derived nutrients exert neuroprotective effects across diverse neurological and neuropsychiatric conditions, endeavoring to elucidate the underlying mechanisms and to advocate for a healthy dietary regimen and judicious intake of dietary constituents to mitigate the risk of neurological disorders and enhance neurological wellbeing.

## 1 Convergent mechanisms: neuroinflammation, oxidative stress, and ferroptosis

Multiple articles converge on the Nrf2/GPX4 axis as a critical defense against ferroptosis, an iron-dependent, lipid peroxidation-driven cell death pathway implicated in AD, PD, and other neurodegenerative disorders. Hong et al. demonstrate that culinary spices (curcumin, allicin, cinnamaldehyde, capsaicin, and piperine) function as natural ferroptosis inhibitors through electrophilic modification of Keap1, releasing Nrf2 to activate GPX4. Curcumin additionally chelates iron, while piperine co-administration increases curcumin bioavailability up to 2,000%. Complementing this, Zhang L. et al. review how herbal compounds (baicalein, curcumin, astragaloside IV) modulate neuroinflammation through TLR4/NF-κB, NLRP3 inflammasome, and JAK-STAT pathways, while promoting microglial M1-to-M2 and astrocytic A1-to-A2 phenotypic shifts. Lu et al. detail quercetin's multi-target cerebrovascular protection via Nrf2/HO-1 activation, Wnt/β-catenin-mediated BBB stabilization, and BDNF-TrkB-PI3K/Akt anti-apoptosis, while candidly acknowledging the “delivery gap” (oral bioavailability < 6%). Zhong et al. review the antidepressant effects of culinary spices, emphasizing “food synergy”—curcumin with piperine—and multi-pathway actions including monoamine regulation, HPA axis inhibition, and BDNF activation. Tang et al. experimentally validate Danggui Shaoyao San, demonstrating that this medicinal-food formula exerts antidepressant effects through coordinated TLR4/NF-κB, JAK2/STAT3, and AKT-GSK3β modulation, promoting hippocampal neurogenesis. Li L. et al. employ machine learning and network pharmacology to identify daidzein from Pueraria as an antidepressant compound targeting CDK5R1 (binding energy −8.3 kcal/mol). Wang Q. et al. show that MgSO4 supplementation alleviates CMS-induced depression by suppressing IKK/NF-κB and NLRP3 inflammasome signaling, reducing microglial activation, and repairing BBB integrity—linking mineral nutrition directly to neuroinflammation control. Liu G. et al. bridge neuroimaging with nutrition, demonstrating that recurrent MDD patients show bilateral amygdala dysfunction and reduced BDNF levels, with BDNF positively correlating with amygdala functional connectivity.

## 2 Alzheimer's and Parkinson's disease: lipid metabolism and proteomic insights

Wang Y. et al. use quantitative proteomics in 5 × FAD mice to show that Modified Sanjia Powder (containing six medicine-food homology components) downregulates ACSL4, a key regulator linking lipid metabolism to ferroptosis, reduces Aβ plaques and tau pathology, and improves cognitive function. Ye et al. identify PFKFB3 as a therapeutic target for Pueraria in AD, showing that daidzein and formononetin bind PFKFB3 (energy < −8.0 kcal/mol) and reverse Aβ_1 − 42_-induced cognitive deficits. Wang M. et al. synthesize evidence for medicine-food homology compounds in PD, detailing PD-specific oxidative stress (dopamine auto-oxidation, neuromelanin-iron catalysis) and proposing a quality standardization framework including function-linked release assays (Nrf2/ARE reporter, iPSC-derived TH^+^ neuron protection).

## 3 Cerebrovascular protection and the gut-brain axis

Jin et al. comprehensively review phytochemical strategies for BBB protection across VCI etiologies (stroke, hypertension, diabetes, and aging), identifying five core mechanisms: tight junction restoration, MMP inhibition, neuroinflammation mitigation, oxidative stress alleviation, and gut-brain axis modulation. Yu et al. apply multi-omics integration to Xianyu Capsule, revealing a “gut microbiota-glycerophospholipid metabolism-neuroinflammation” axis in epilepsy treatment.

## 4 Special populations: thyroid disease, chemotherapy, and women's health

Zhang Z. et al. addressed persistent neuropsychiatric symptoms in thyroid disease by linking them to specific dietary components and their underlying mechanisms: selenium through its cofactor role in deiodinases, iodine via its characteristic U-shaped risk curve, the Mediterranean diet by reducing neuroinflammation and oxidative stress, and cruciferous vegetables by the trade-off between neuroprotective sulforaphane and goitrogenic compounds. Li W. et al. systematically review the clinical evidence for plant food-derived nutrients in preventing CIPN. This includes a landmark Level I trial for curcumin, alongside promising findings for green tea catechins (notably EGCG), vitamin E, omega-3 fatty acids, carotenoids, and anthocyanins. Liu X. et al. review plant-derived compounds for women's reproductive health, covering polyphenols (EGCG, curcumin, and resveratrol) and phytoestrogens (isoflavones, lignans). They detail mechanisms targeting the HPO axis and neuroinflammation (NF-κB, Nrf2), applications in menopausal cognition, PMS, and endometriosis, as well as formulation strategies for improved bioavailability.

## 5 Conclusion

The articles in this Research Topic collectively demonstrate that plant-derived nutrients are mechanistically sophisticated, multi-target neuroprotective agents converging on Nrf2/ARE, NF-κB/NLRP3, BDNF-TrkB, and AKT-GSK3β pathways. While bioavailability bottlenecks and the need for large-scale clinical trials remain challenges, the integration of traditional dietary wisdom with modern formulation science and precision nutrition offers a transformative roadmap for neurological health.
